# Supplementary material for: The telomere binding protein Pot1 maintains haematopoietic stem cell activity with age
Source: Nat Commun. 2017 Oct 6;8:804. doi: 10.1038/s41467-017-00935-4 (PMC5630588; doi:10.1038/s41467-017-00935-4)
Supplement: Supplementary file 1 — Supplementary information [file 41467_2017_935_MOESM1_ESM.pdf]

Supplementary Table 1. Result of image analysis

			COLOCALIZATION OF GREEN CHANNEL WITH RED						COLOCALIZATION OF RED CHANNEL WITH GREEN					
	STAINING		MEAN		SD				MEAN		SD			
Samples	GREEN	RED	observed	randomized	observed	randomized	p-value	Welch's t-test p-value	observed	randomized	observed	randomized	p-value	Welch's t-test p-value
8 week-old HSCs	TRF1	53BP1	0.4661	0.3681	0.0525	0.0516	0.0153	<0.0001	0.5783	0.4533	0.048	0.0519	0.0102	<0.0001
90 week-old HSCs	TRF1	53BP1	0.1787	0.1148	0.0306	0.0246	0.0063	<0.0001	0.6255	0.4137	0.0588	0.0635	0.0044	<0.0001
10 days cultured 8 week-old HSCs/Control	TRF1	53BP1	0.7653	0.6533	0.0273	0.0318	0.0002	<0.0001	0.2244	0.1834	0.0156	0.0151	0.0002	<0.0001
10 days cultured 8 week-old HSCs/MTM-POT1a treated	TRF1	53BP1	0.6012	0.4609	0.0321	0.0337	<0.0001	<0.0001	0.2069	0.1454	0.0172	0.014	<0.0001	<0.0001
10 days cultured 90 week-old HSCs/Control	TRF1	53BP1	0.5819	0.5078	0.029	0.0286	0.0064	<0.0001	0.2524	0.213	0.0168	0.0161	0.0045	<0.0001
10 days cultured 90 week-old HSCs/MTM-POT1a treated	TRF1	53BP1	0.7512	0.6388	0.0231	0.028	<0.0001	<0.0001	0.2442	0.2009	0.0136	0.0124	<0.0001	<0.0001
3 week cultured 8 week-old HSCs/Control	TRF1	53BP1	0.4991	0.3198	0.0327	0.0321	<0.0001	<0.0001	0.4519	0.2893	0.0262	0.0265	<0.0001	<0.0001
3 week cultured 8 week-old HSCs/MTM-POT1a treated	TRF1	53BP1	0.5338	0.348	0.0405	0.041	<0.0001	<0.0001	0.3197	0.2071	0.0282	0.026	<0.0001	<0.0001
Control-shRNA transduced HSCs	TRF1	53BP1	0.2913	0.24	0.0578	0.0558	0.1653	<0.0001	0.4524	0.3741	0.0685	0.07	0.171	<0.0001
shPot1a transduced HSCs	TRF1	53BP1	0.2486	0.2009	0.0576	0.0532	0.1966	<0.0001	0.3444	0.2786	0.0671	0.0636	0.2014	<0.0001
Control GFP transduced HSCs /4M post BMT	TRF1	53BP1	0.5822	0.4229	0.1305	0.1353	0.1633	<0.0001	0.1326	0.0949	0.0442	0.0391	0.1785	<0.0001
Pot1a transduced HSCs /4M post BMT	TRF1	53BP1	0.6963	0.5256	0.1352	0.1525	0.1645	<0.0001	0.135	0.0951	0.0563	0.0426	0.1847	<0.0001
10 days cultured human CB HSCs /Control	TRF1	53BP1	0.509	0.4368	0.0358	0.0352	0.0092	<0.0001	0.4992	0.4229	0.0313	0.0313	0.0081	<0.0001
10 days cultured human CB HSCs /MTM-hPOT1 treated	TRF1	53BP1	0.4704	0.3174	0.0357	0.0342	<0.0001	<0.0001	0.3613	0.2495	0.0306	0.0281	0.0007	<0.0001
10 days cultured human CB HSCs /Control	TRF1	RPA32	0.3642	0.228	0.0322	0.0284	0.0001	<0.0001	0.3995	0.246	0.033	0.0287	<0.0001	<0.0001
10 days cultured human CB HSCs /MTM-hPOT1 treated	TRF1	RPA32	0.1494	0.1045	0.0217	0.018	0.0061	<0.0001	0.5428	0.4475	0.0552	0.0541	0.0906	<0.0001

Supplementary Table 2. TaqMan® Gene Expression Assay Mixes for Q-PCR analysis

Gene name	Gene symbol	Assay ID
Acd (Tpp1)	*Acd*	Mm01247801_g1
Ataxia telangiectasia mutated homolog	*Atm*	Mm01177457_m1
BCL2-associated agonist of cell death	*Bad*	Mm00432042_m1
BCL2-antagonist/killer 1	*Bak1*	Mm00432045_m1
BCL2-associated X protein	*Bax*	Mm00432050_m1
BCL2 binding component 3 (PUMA)	*Bbc3*	Mm00519268_m1
B cell leukemia/lymphoma 2	*Bcl2*	Mm00477631_m1
Bmi1 polycomb ring finger oncogene	*Bmi1*	Mm00776122_gH
CD34 antigen	*Cd34*	Mm00519283_m1
CD48 antigen	*Cd48*	Mm00455932_m1
Cyclin-dependent kinase inhibitor 1A (P21)	*Cdkn1a*	Mm00432448_m1
Cyclin-dependent kinase inhibitor 1C (P57)	*Cdkn1c*	Mm01272135_g1
Colony stimulating factor 1 receptor	*Csf1r*	Mm00432689_m1
Colony stimulating factor 3 receptor	*Csf3r*	Mm00432735_m1
Cyclin-dependent kinase inhibitor 2A (Cdkn2a), transcript variant 1	*p19Arf*	Custom*
Cyclin-dependent kinase inhibitor 2A (Cdkn2a), transcript variant 2	*p16Ink4a*	Custom**
Chemokine (C-X-C motif) receptor 4	*Cxcr4*	Mm01292123_m1
Enhancer of zeste homolog 2	*Ezh2*	Mm00468464_m1
FMS-like tyrosine kinase 3	*Flt3*	Mm00438996_m1
Forkhead box O1	*Foxo1*	Mm00490672_m1
Forkhead box O3	*Foxo3*	Mm01185722_m1
GATA binding protein 2	*Gata2*	Mm00492300_m1
Hairy and enhancer of split 1	*Hes1*	Mm01342805_m1
Homeobox B4	*Hoxb4*	Mm00657964_m1
Interferon regulatory factor 8	*Irf8*	Mm00492567_m1
MDS1 and EVI1 complex locus (Evi1)	*Mecom* (*Evi1*)	Mm00514814_m1
Myeloproliferative leukemia virus oncogene	*Mpl*	Mm00440310_m1
Myelocytomatosis oncogene	*Myc*	Mm00487803_m1

v-myc myelocytomatosis viral related oncogene, neuroblastoma derived	*Mycn*	Mm00627179_m1
Necdin	*Ndn*	Mm02524479_s1
Protection of telomeres 1A	*Pot1a*	Mm00505816_m1
Protection of telomeres 1B	*Pot1b*	Mm01278790_m1
Protein C receptor, endothelial	*Procr*	Mm00440992_m1
Selectin, platelet	*Selp*	Mm00441295_m1
Signaling lymphocytic activation molecule family member 1	*Slamf1*	Mm00443316_m1
T cell acute lymphocytic leukemia 1	*Tal1*	Mm00441665_m1
Endothelial-specific receptor tyrosine kinase (Tie2)	*Tek*	Mm00443242_m1
Telomerase RNA component	*Terc*	Mm01261365_s1
Telomeric repeat binding factor 1	*Terf1*	Mm00436923_m1
Telomeric repeat binding factor 2	*Terf2*	Mm01253555_m1
Telomeric repeat binding factor 2, interacting protein (Rap1)	*Terf2ip* (*Rap1*)	Mm01243676_m1
Telomerase reverse transcriptase	*Tert*	Mm01352136_m1
Terf1 (TRF1)-interacting nuclear factor 2	*Tinf2*	Mm00461166_g1
Transformation related protein 53	*Trp53*	Mm00441964_g1
Mechanistic target of rapamycin (serine/threonine kinase)	*Mtor*	Mm00444968_m1
regulatory associated protein of MTOR, complex 1	*Rptor*	Mm01242613_m1
Mouse ACTB (actin, beta) endogenous control	*Actb*	4352933E
Protection of telomeres 1 (human POT1)	*POT1*	Hs00209984_m1
Human ACTB (actin, beta)	*ACTB*	Hs01060665_g1

Supplementary Table 3. Primer and probe sequence for p16Ink4a and p19Arf

p19Arf	Forward	GTCACACGACTGGGCGATT
	Reverse	CAGTCTGTCTGCAGCGGACTC
	Probe	FAM-CACTGAATCTCCGCGAGG-MGB
p16Ink4a	Forward	GGGTTTTCTTGGTGAAGTTCGT
	Reverse	AAGATCCTCTCTAGCCTCAACAACA
	Probe	FAM-ACAGCGAGCTGCGCT-MGB

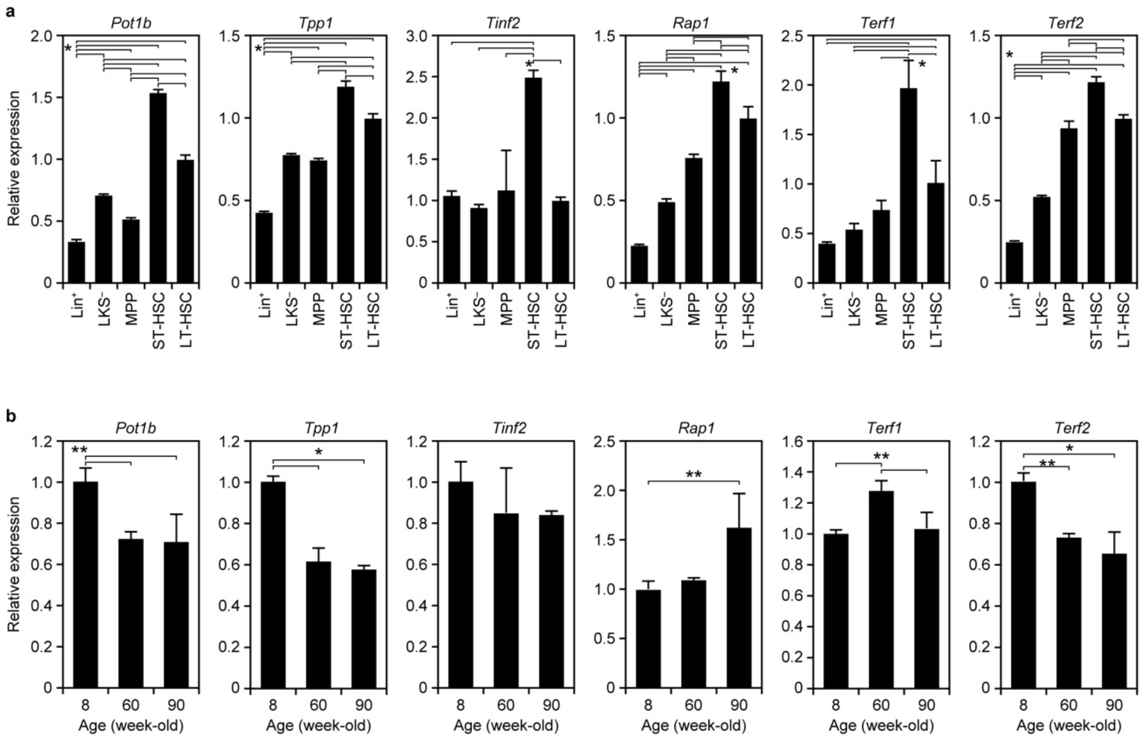

Supplementary Figure 1. Expression of shelterin components in fractionated hematopoietic cells

(a) Expression of *Pot1b*, *Tpp1*, *Tinf2*, *Rap1*, *Terf1*, and *Terf2* in fractionated hematopoietic cells isolated from 8 week-old mice. Lin^{+}, $lineage^{+}$ cells; LKS^{-} cells, $Lin^{-}Kit^{+}Sca\text{-}1^{-}$ cells; MPP, $LSKCD41^{+}CD48^{+}CD150^{-}$ cells; $LSKCD41^{+}CD48^{+}CD150^{+}$ ST-HSCs; $LSKCD41^{-}CD48^{-}CD150^{+}$ LT-HSCs. Data are expressed as the mean ± SD (n = 4, *p < 0.05 by Tukey's test). Representative data from 3 independent experiments are shown. (b) Expression of *Pot1b*, *Tpp1*, *Tinf2*, *Rap1*, *Terf1*, and *Terf2* in 8, 60 and 90 week-old LT-HSCs. Data are expressed as the mean ± SD (n = 4, *p < 0.01, **p < 0.05 by Tukey's test). Representative data from two independent experiments are shown.

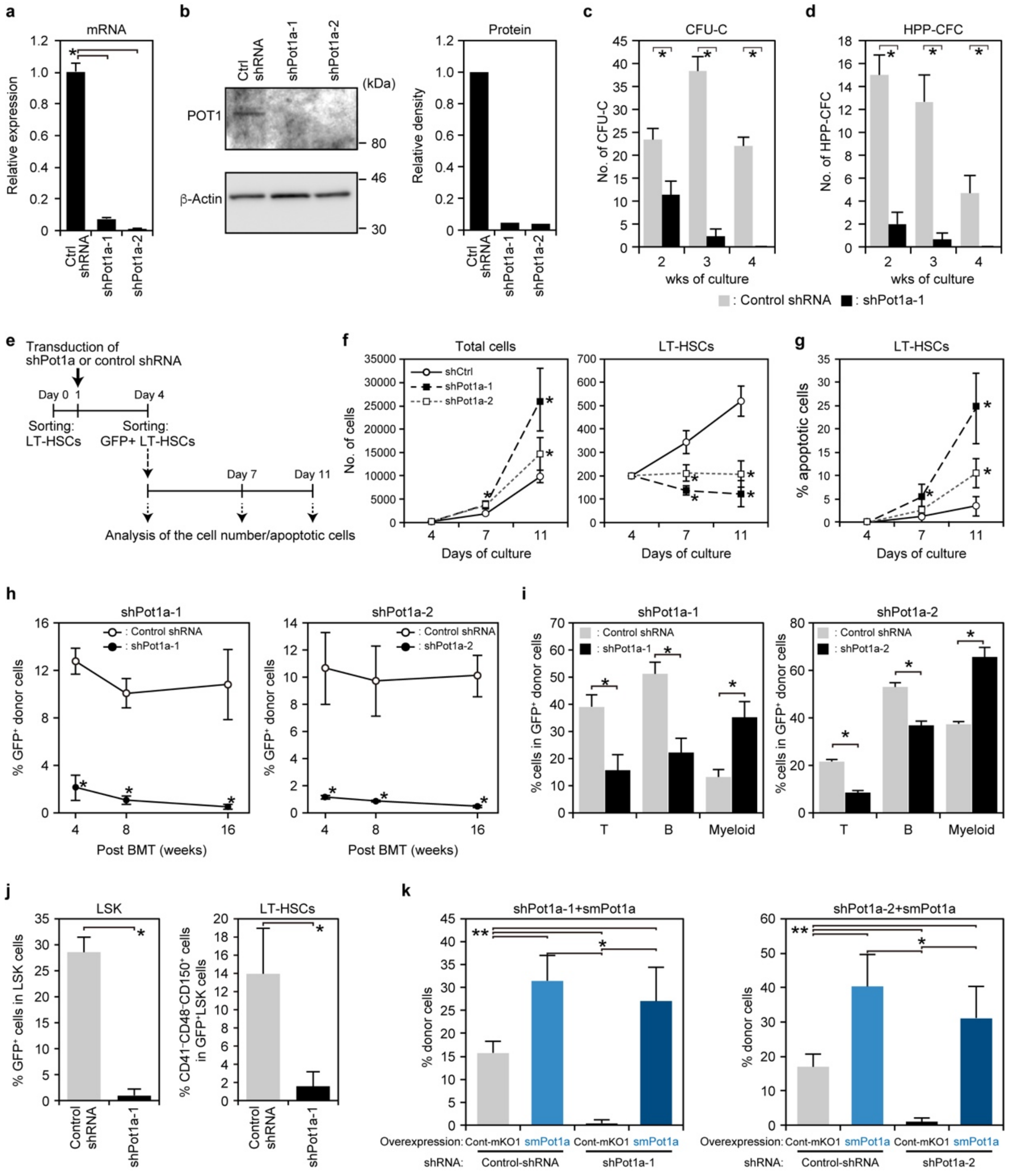

Supplementary Figure 2. Knockdown of Pot1a impairs HSC function.

(a, b) Efficiency of the knockdown of Pot1a. LT-HSCs (8 week-old) were transduced control shRNA, shPot1a-1 and -2. After 2 days of transduction, GFP^{+} LT-HSCs cells were collected the expression of Pot1a mRNA and POT1 protein was analyzed. (a) Q-PCR analysis of the expression of *Pot1a*. Data represent the mean ± SD (*p < 0.01 by Tukey's test). (b) Western blot analysis of POT1 (left panel). Densitometry analysis of the western blot is shown in the right panel. (c, d) Control shRNA or shPot1a-1-transduced 8 week-old LSK cells were cultured for 2–4 weeks, and

colony formation was analyzed. (c) Number of CFU-Cs. (d) Number of HPP-CFCs. Data are expressed as the mean ± SD (n = 3, *p < 0.01 by *t*-test).

(e) Schematic of the analysis of cell number and apoptosis of LT-HSCs after the knockdown of Pot1a. (f) Number of total cells and LT-HSCs on day 4, 7, and 10 of culture. Data are expressed as the mean ± SD (n = 8-9, *p < 0.01 by *t*-test). (g) Percentage of Annexin V^+PI^+ apoptotic cells in LT-HSCs on day 4, 7, and 11 of culture. Data are expressed as the mean ± SD (n = 6-7, *p < 0.01 by *t*-test).

(h-j) Effects of Pot1a knockdown on HSC LTR activity. (h) Percentages of GFP^+ donor-derived ($Ly5.1^+$) cells in recipient mice peripheral blood (PB) after 4, 8, and 16 weeks BMT. Data are expressed as the mean ± SD (n = 7/group, *p < 0.01 by *t*-test) from 3 independent experiments for shPot1a-1 and -2. (i) Percentage of B cells ($B220^+$), T cells ($CD3^+$), and myeloid ($Mac\text{-}1^+/Gr\text{-}1^+$) cells in GFP^+ donor-derived cells 4 months after BMT. Data are expressed as the mean ± SD (n = 7, *p < 0.01 by *t*-test). (j) Percentage of GFP^+ donor cells in BM LSK and LT-HSCs.

(k) LT-HSCs were co-transduced with a retrovirus expressing shPot1a-1 or -2 and a retrovirus expressing control-monomeric Kusabira-Orange1 (mKO1) or smPot1a-mKO1. After retroviral transduction, LT-HSCs (control-shRNA$^+$/control-mKO1$^+$, control-shRNA$^+$/smPot1a$^+$, shPot1a$^+$/control-mKO1$^+$, and shPot1a$^+$/smPot1a$^+$) were isolated and transplanted into lethally irradiated recipient mice. Percentage of donor-derived cells in PB 4 months after BMT is shown. Data are expressed as the mean ± SD (n = 5/group, *p < 0.01 **p<0.05 by Tukey's test).

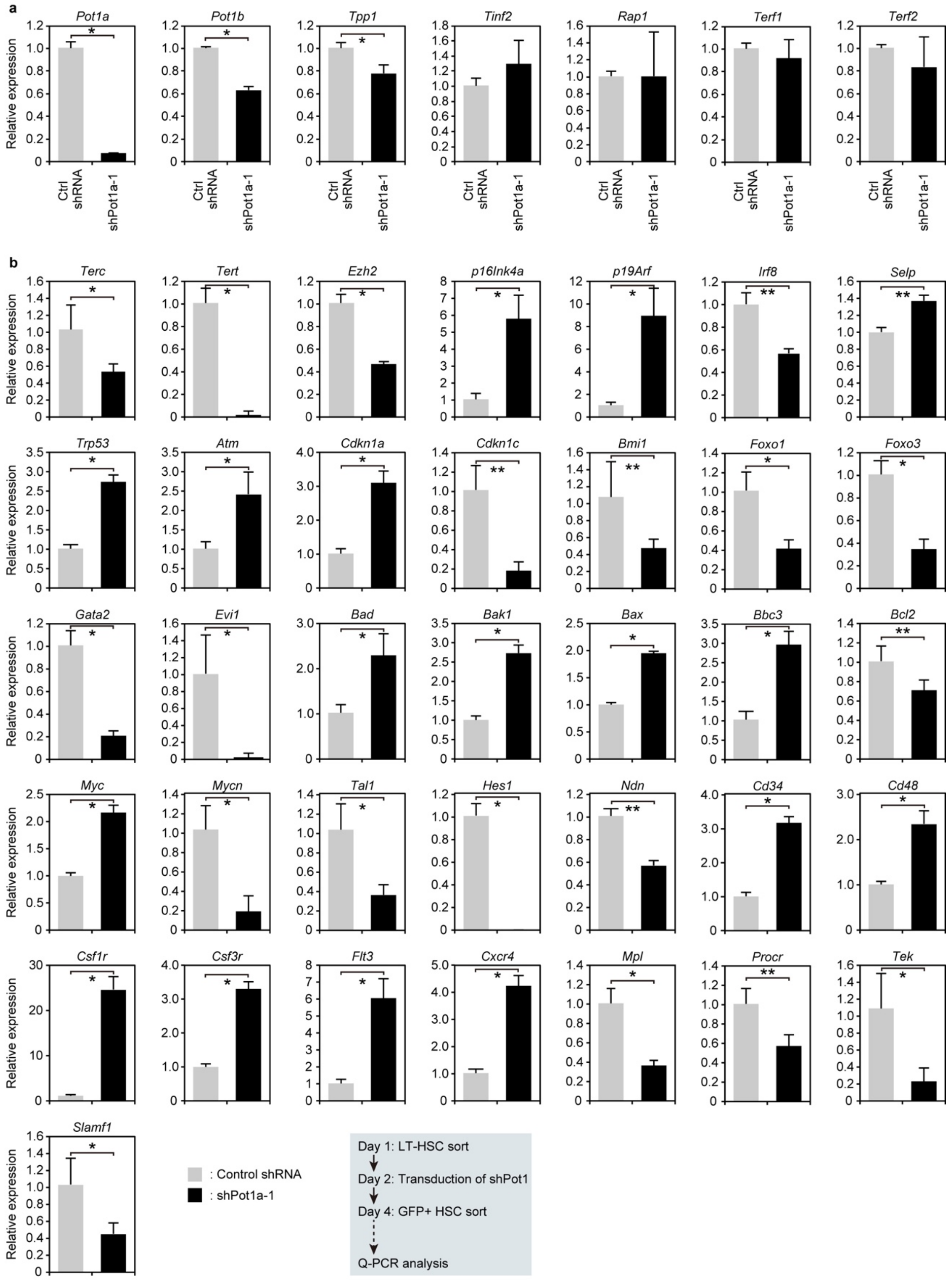

Supplementary Figure 3. Effects of *Pot1a* KD on HSC gene expression patterns.

LSKCD41^{-}CD48^{-}CD150^{+} LT-HSCs were transduced with shPot1a or control shRNA. After transduction of the shRNAs, GFP^{+} HSCs were sorted and gene expression patterns were analyzed.

(a) Expression of shelterin component genes in control shRNA or shPot1a-1 transduced HSCs. Data are expressed as the mean ± SD (n = 4, *p < 0.01 by *t*-test). (b) shPot1a-transduced cells showed significantly lower expression levels of *Tpp1*, *Tert*, and *Terc*; the anti-apoptotic gene *Bcl2*; senescence-related genes *Ezh2* and *Irf8*; cell cycle-related genes *Cdkn1c*, *Foxo1*, *Foxo3a*, *Mycn*, and *Ndn*; and significantly higher levels of the pro-apoptotic genes *Bad*, *Bak1*, *Bax*, and *Bbc3*; cell cycle/DNA damage response-related genes *Atm*, *Cdkn1a*, and *Trp53*; and senescence-related genes including *p19Arf*, *p16Ink4a*, and *Selp*. shPot1a-transduced LSK cells also showed significantly lower expression of HSC maintenance-related/marker genes, and higher expression of differentiation markers than control shRNA-transduced LSK cells. Data are expressed as the mean ± SD (n = 6, *p < 0.01, **p < 0.05 by *t*-test). The bottom right panel shows a schematic of the experimental procedure.

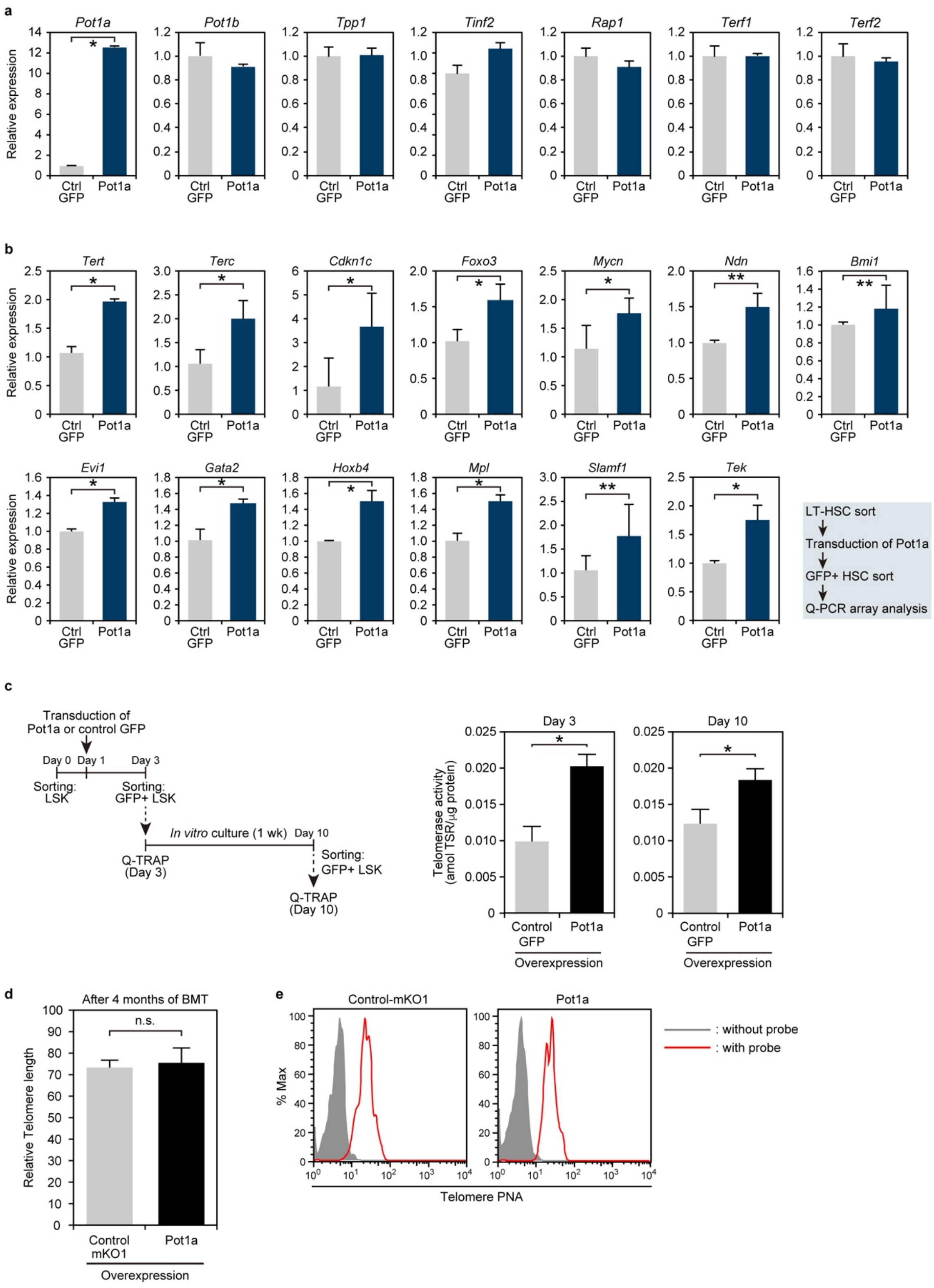

Supplementary Figure 4. Effects of Pot1a overexpression on HSC gene expression patterns and telomerase activity.

LSKCD41$^-$CD48$^-$CD150$^+$ LT-HSCs were transduced with control GFP or Pot1a. After retroviral transduction, GFP$^+$ LSK cells were sorted and gene expression patterns were analyzed. (a) Expression of shelterin component genes after overexpression of Pot1a. Data are expressed as the mean ± SD (n = 4, *p < 0.01 by *t*-test) (b) Overexpression of Pot1a induced the upregulation of *Tert*, *Terc*, *Cdkn1c*, *Foxo3*, *Mycn*, *Ndn*, *Bmi1*, *Evi1*, *Gata2*, *Hoxb4*, *Mpl*, *Slamf1*, and *Tek* (*Tie2*). Data are expressed as the mean ± SD (n = 6, *p < 0.01, **p < 0.05 by *t*-test). The bottom right panel shows a schematic of the experimental procedure.

(c-e) Effects of Pot1a overexpression on telomerase activity and telomere length in HSCs. (c) Schematic of the telomerase activity assay (left). LSK cells were transduced with control GFP or Pot1a. After 2 days of retroviral transduction, GFP$^+$ LSK cells were sorted and telomerase activity was measured by a quantitative telomeric repeat amplification protocol (Q-TRAP) method. Simultaneously, sorted GFP$^+$ LSK cells were re-cultured for 1 week. After culture, GFP$^+$ LSK cells were re-isolated and the telomerase activity was measured by Q-TRAP. Results of Q-TRAP assay on day 3 and day 10 are shown in the right panels. Data are expressed as the mean ± SD (n = 4, *p < 0.01 by *t*-test). Representative data from two independent experiments are shown. (d, e) Control mKO1- or Pot1a-transduced LSK cells were transplanted into lethally irradiated recipient mice. 4 months after BMT, mKO1$^+$ donor-derived LSK cells were isolated and telomere length was assessed by Flow FISH. (d) Relative telomere length. Data are expressed as the mean ± SD (n = 4, n.s., not significant by *t*-test). (e) Representative histograms of telomere PNA.

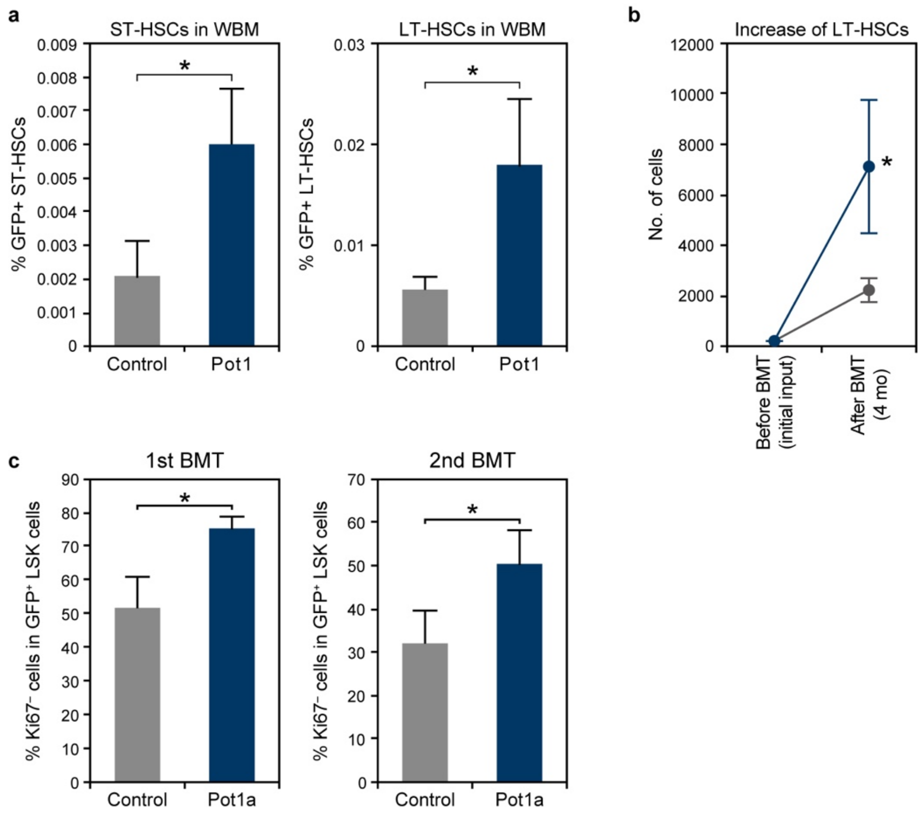

Supplementary Figure 5. Effect of Pot1a overexpression on the number and cell cycle quiescence of LT-HSCs after BMT.

(a) Control GFP or Pot1a-transduced LT-HSCs were transplanted into lethally irradiated mice (250 cells/mice). After 5 month of BMT, the percentage of donor-derived (GFP^+) ST-HSC and LT-HSC in whole BM cells (WBM) was analyzed. (b) Increase of the number of LT-HSCs after BMT. Data are expressed as the mean ± SD (n = 5, *p < 0.01 by *t*-test). Representative data from two independent experiments are shown. (c) Percentage of $Ki67^-$ cells in donor-derived (GFP^+) LSK cells after 1st and 2nd BMT. Data are expressed as the mean ± SD (n = 5: 1st BMT, n = 6: 2nd BMT, *p < 0.01 by *t*-test).

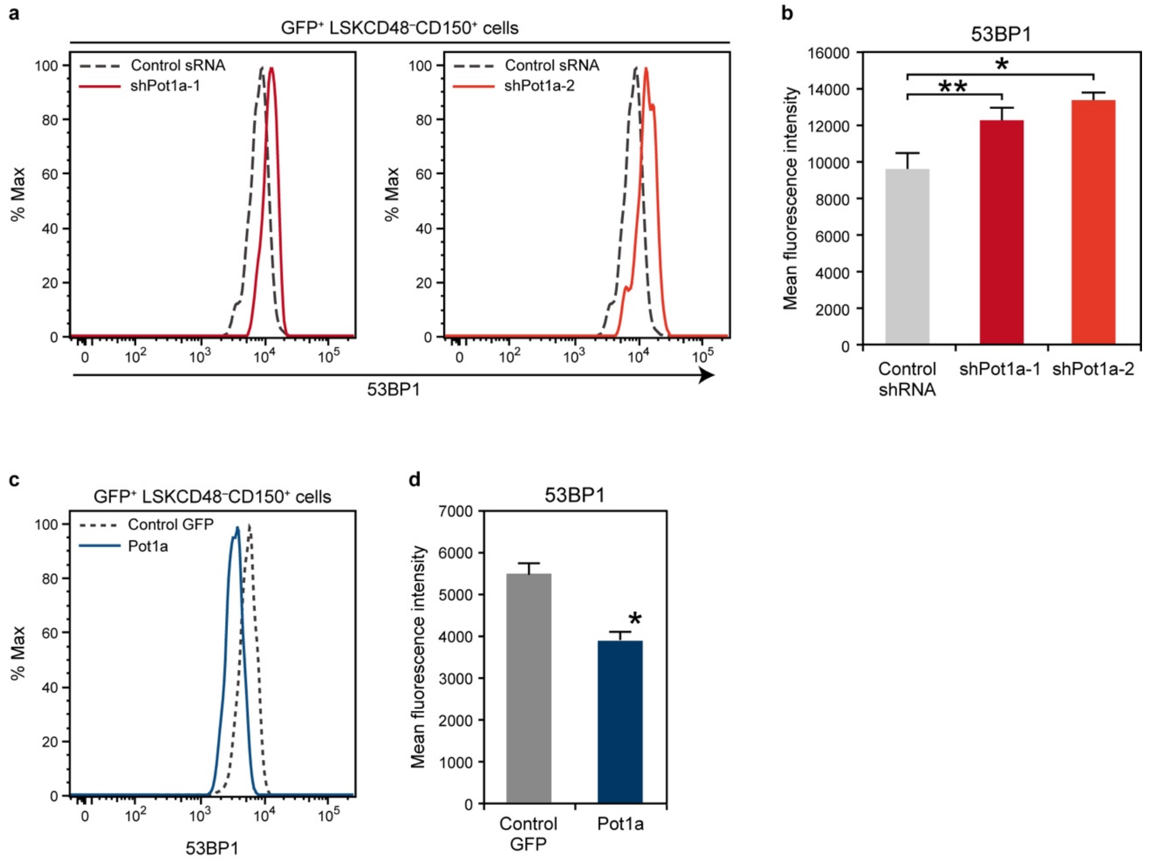

Supplementary Figure 6. 53BP1 expression level in Pot1a-overexpressing or -knockdown LT-HSCs after the culture.

(a, b) LT-HSCs were transduced with shPot1a-1, -2 or control shRNA. After 1 week of culture, 53BP1 level in GFP$^+$LT-HSC fraction was analyzed. (a) Representative FACS profiles of 53BP1. (b) Mean fluorescence intensity of 53BP1. Data are expressed as the mean ± SD (n = 5, *p < 0.01, **p < 0.05 by Tukey's test).

(c, d) LT-HSCs were transduced with Pot1a or control-GFP were cultured for 2 weeks. After the culture, 53BP1 level in gene transduced GFP$^+$LT-HSCs was analyzed. (c) Representative FACS profiles of 53BP1. (d) Mean fluorescence intensity of 53BP1. Data are expressed as the mean ± SD (n = 9, *p < 0.01 by *t*-test).

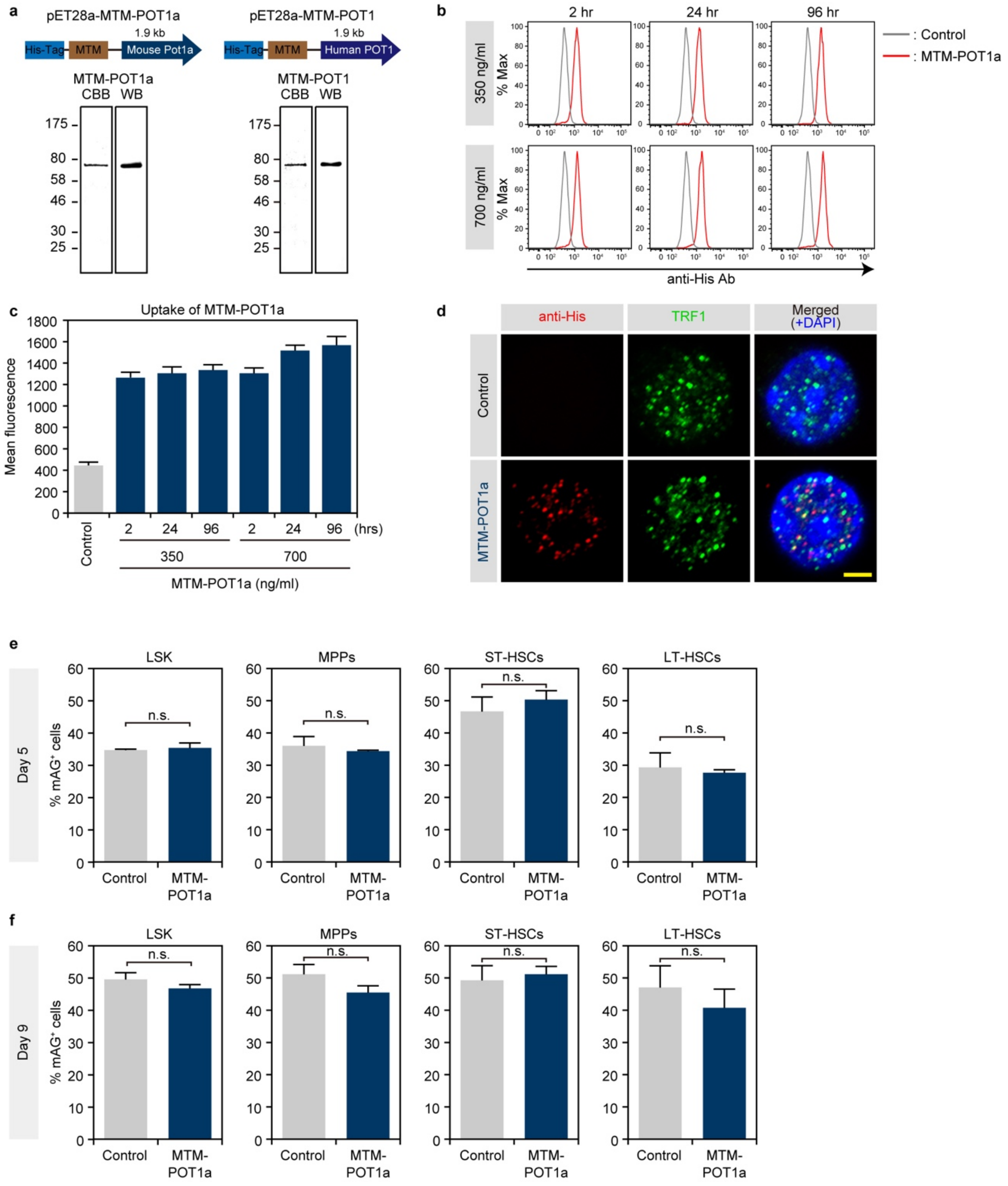

Supplementary Figure 7. Preparation of MTM-POT1a/POT1 proteins.

(a) Vector construction of pET28a-MTM-POT1a (left) and -POT1 (right). Full-length mouse Pot1a or human POT1 was inserted into the multi-cloning site (MCS) of pET28a vector which have a His- and MTM-tag at the N-terminus of MCS (upper). Purification of MTM-proteins (lower). The protein fraction was separated by sodium dodecyl sulfatepolyacrylamide gel electrophoresis and transferred to a polyvinylidene difluoride membrane following purification.

The transferred protein was detected by immunoblotting with an anti-His Ab (right), and the membrane was stained with Coomassie brilliant blue (left). The purified MTM-POT1a migrated as a single band on sodium dodecyl sulfate–polyacrylamide gels stained with Coomassie brilliant blue. The apparent mobility of the tagged protein corresponded to the expected size of 74 kDa. (b) Representative FACS profiles of anti-His staining of LT-HSCs cultured with 350 ng/ml (upper) and 700 ng/ml (lower) of MTM-POT1a. The majority of LT-HSCs incorporated MTM-POT1a within 2 hours. (c) Relative mean fluorescent intensity of LT-HSCs cultured with 350 ng/ml (upper) and 700 ng/ml (lower) of MTM-POT1a. Data are expressed as the mean ± SD (n = 3). (d) The localization of recombinant POT1a in LT-HSCs was confirmed by immunocytochemistry. LT-HSCs cultured with MTM-POT1a overnight were co-stained with anti-TRF1 (green) and anti-His-Tag (red) Abs, and the nuclei were detected by DAPI (blue). Scale bar, 2 μm.
(e, f) Function of MTM-POT1a in cell cycle progression (%S/G2/M) of HSPCs. LT-HSCs from Fucci mice were cultured for 9 days with or without MTM-POT1a (350 ng/ml). After 5 (e) and 9 (f) days of culture, the percentage of monomeric Azami-Green 1-positive (mAG^+) cells, corresponding to the percentage of cells in S/G2/M phase, in LSK, MPP, ST-HSC, and LT-HSC fractions was analyzed. MTM-POT1a did not affect the frequency of S/G2/M cells in any of the cultures. Data are expressed as the mean ± SD (n = 3. n.s., not significant by *t*-test).

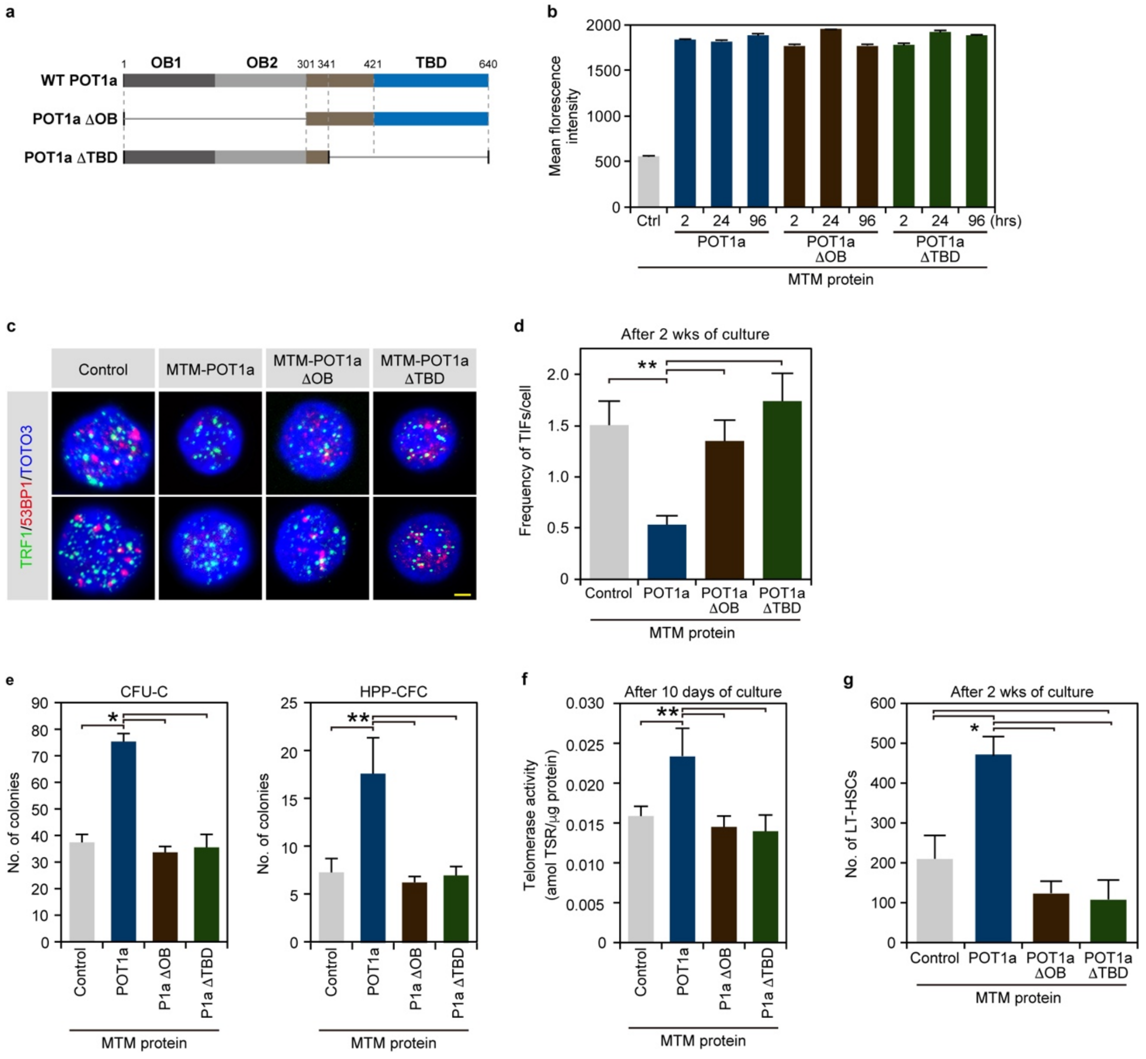

Supplementary Figure 8. Effect of mutant forms of POT1a on HSC function.

(a) Schematic of wild type (full-length) POT1a and mutant forms of POT1a lacking OB-fold domains (POT1aΔOB) or Tpp1 biding domain (TBD) (POT1aΔTBD). (b) Relative mean fluorescent intensity of LT-HSCs cultured with control MTM-protein, wild type MTM-POT1a, MTM-POT1aΔOB and MTM-POT1aΔTBD. Data are expressed as the mean ± SD (n = 3). (c, d) Effect of mutant forms of POT1a on the prevention of telomeric DDR in LT-HSCs during culture. (c) Immunocytochemical staining of TRF1 (green) and 53BP1 (red). Scale bar, 2 μm. (d) Frequencies of TIFs in LT-HSCs after 2 weeks of culture are shown. Data are expressed as the mean ± SD (n = 22-25, **p < 0.05 by Tukey's test). (e) Effect of mutant forms of POT1a MTM protein on the colony forming ability. Data are expressed as the mean ± SD (n = 3, *p < 0.01 by Tukey's test). Representative data from two independent experiments are shown. (f) Effect of wild-type and mutant forms of POT1a on the telomerase activity of LT-HSCs. LT-HSCs were

cultured for 10 days with MTM proteins and the telomerase activity was measured by Q-TRAP. Data are expressed as the mean ± SD ($n = 4$, *$p < 0.01$ by Tukey's test). Representative data from two independent experiments are shown. (g) Effect of MTM proteins on the expansion of LT-HSCs *in vitro*. LT-HSCs (200 cells/well) were cultured with MTM-POT1a or mutant forms of POT1a for 2 weeks. Numbers of LT-HSCs after culture are shown. Data are expressed as the mean ± SD ($n = 4$, *$p < 0.01$ by Tukey's test). Representative data from two independent experiments are shown.

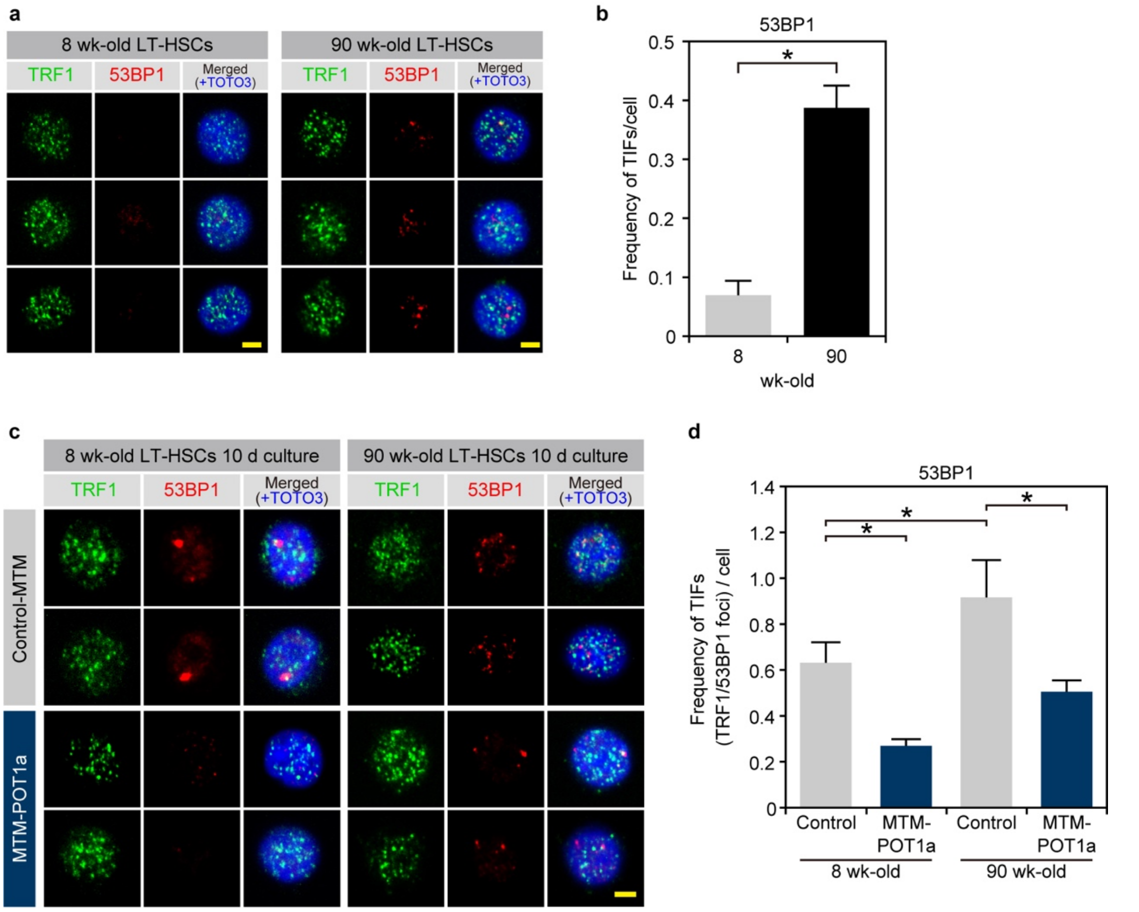

Supplementary Figure 9. Effect of MTM-POT1a on the prevention of DNA damage in aged LT-HSCs.

(a) Immunocytochemical staining of TRF1 (green) and 53BP1 (red) in LT-HSCs isolated from 8- and 90-week-old mice. Nuclei were stained with TOTO-3 (blue). Scale bar, 2 µm. (b) Frequency of TIFs per cell. Data are expressed as the mean ± SD (n = 90-100, *p < 0.01 by *t*-test). Representative data from three independent experiments are shown. (c, d) DNA damage response in LT-HSCs isolated from 8- and 90-week-old mice after *in vitro* culture. LT-HSCs were cultured for 10 days with control MTM protein or MTM-POT1a (350 ng/ml) and stained with anti-TRF1, and -53BP1 Abs. Nuclei stained with TOTO-3 (blue). (c) Immunocytochemical staining of TRF (green) and 53BP1 (red). Scale bar, 2 µm. (d) Frequency of TIFs in 8- and 90-week-old LT-HSCs after 10 days of culture. Data are expressed as the mean ± SD (n = 80–100, *p < 0.01 by Tukey's test). Representative data from 2 independent experiments are shown.

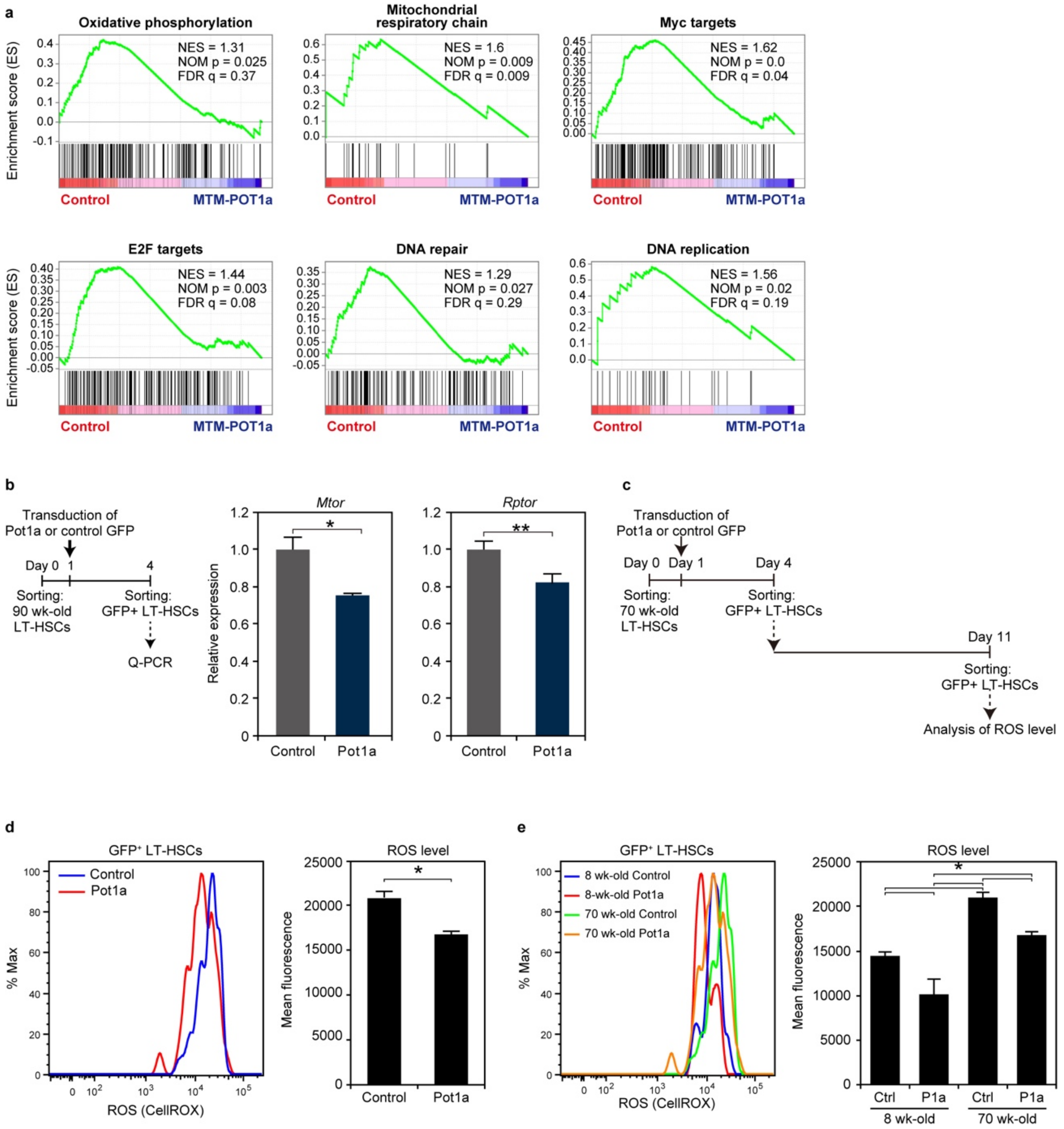

Supplementary Figure 10. Effect of exogenous Pot1a on the gene expression and ROS production in aged LT-HSCs.

(a) LT-HSCs (60 week-old) were cultured with MTM-POT1a or control MTM protein for 3 days. After the culture, gene expression profiles were analyzed by microarray. GSEA plots demonstrating enrichment levels of indicated gene sets in control MTM protein treated cells versus MTM-POT1a treated cells. NES, NOM P value, and FDR are indicated.

(b) Aged LT-HSCs (90 week-old) were transduced with control GFP or Pot1a. After 3 days post-retrovirus transduction, GFP^{+}LT-HSCs were isolated and the gene expression was examined by Q-PCR. Expression of *Mtor* and *Rptor* in Pot1a-overexpressing 90 week-old LT-HSCs was shown.

Data are expressed as the mean ± SD (n = 4, *p < 0.01 by *t*-test).

(c) Schematic of the measurement of ROS in aged LT-HSCs. Control-GFP and Pot1a-transduced LT-HSCs were cultured for 3 days. After the culture, GFP^+ LT-HSCs were isolated and re-cultured for 7 days. After the culture, intracellular ROS was measured. (d) Intracellular ROS levels in control-GFP (blue) and Pot1a-transduced (red) LT-HSCs. Representative FACS profiles of CellROX® in GFP^+ LT-HSCs were shown in left panel. Mean fluorescence level was shown in right panel. Data are expressed as the mean ± SD (n = 5, *p < 0.01 by *t*-test). (e) Comparison of ROS levels between cultured young and aged LT-HSCs that transduced with control-GFP and Pot1a. Representative FACS profiles was shown in left panel. Mean fluorescence level was shown in right panel. Data are expressed as the mean ± SD (n = 5, *p < 0.01 by Tukey's test).

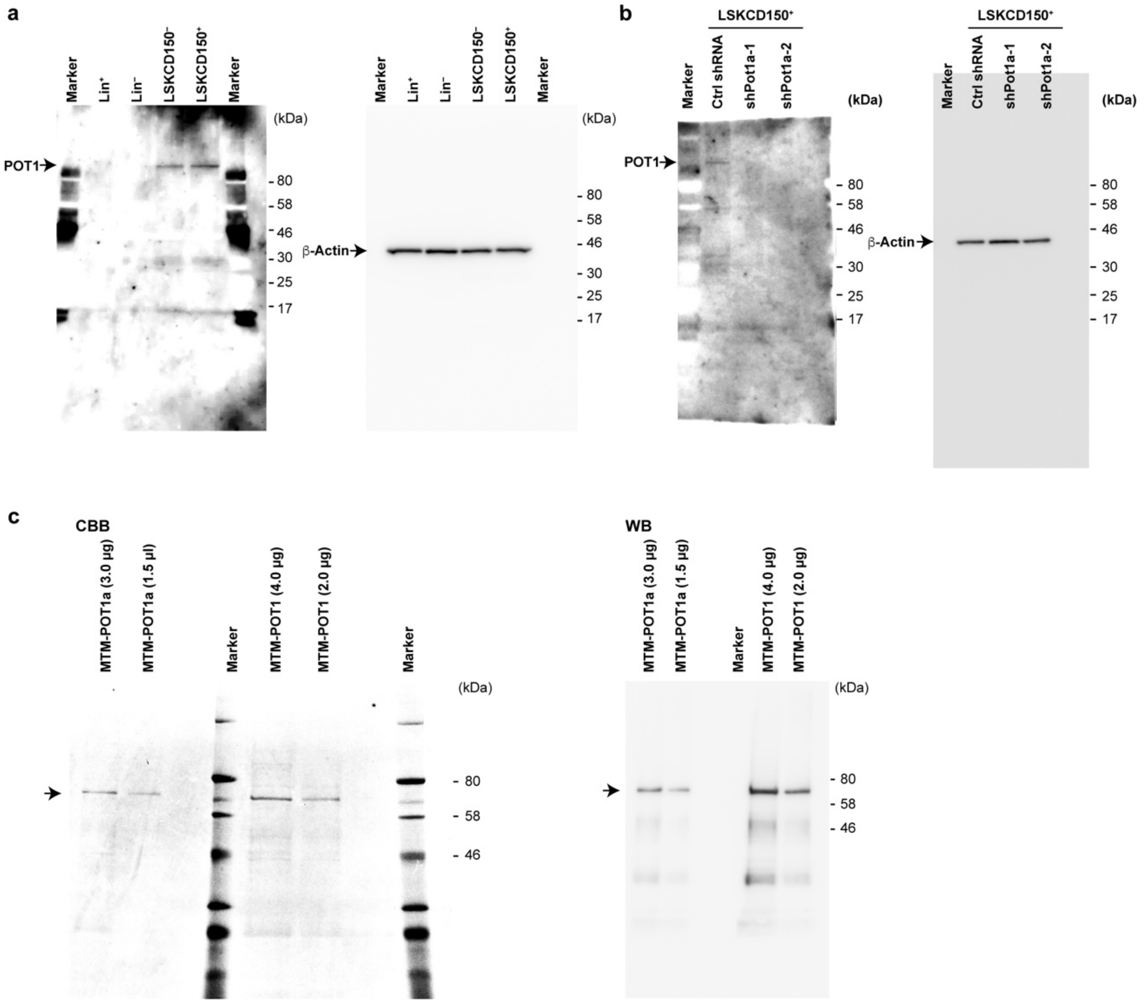

Supplementary Figure 11. Uncropped images of western blots and CBB.

(a) POT1 and β-Actin shown in Fig. 1d. (b) POT1 and β-Actin shown in Supplementary Fig. 2b. (c) CBB staining (left panel) and western blot (right panel) of MTM-POT1a/POT1 shown in Supplementary Fig. 7a.
